# Evaluation of Multiband EPI Acquisitions for Resting State fMRI

**DOI:** 10.1371/journal.pone.0136961

**Published:** 2015-09-16

**Authors:** Christine Preibisch, J. Gabriel Castrillón G., Martin Bührer, Valentin Riedl

**Affiliations:** 1 Department of Neuroradiology, Technische Universität München, Munich, Germany; 2 Clinic for Neurology, Technische Universität München, Munich, Germany; 3 Institute for Biomedical Engineering, University and ETH Zurich, Zurich, Switzerland; 4 GyroTools, Zurich, Switzerland; 5 Clinic for Nuclear Medicine, Technische Universität München, Munich, Germany; University of Maryland, College Park, UNITED STATES

## Abstract

Functional magnetic resonance imaging (fMRI) and particularly resting state fMRI (rs-fMRI) is widely used to investigate resting state brain networks (RSNs) on the systems level. Echo planar imaging (EPI) is the state-of-the-art imaging technique for most fMRI studies. Therefore, improvements of EPI might lead to increased sensitivity for a large amount of studies performed every day. A number of developments to shorten acquisition time have been recently proposed and the multiband technique, allowing the simultaneous acquisition of multiple slices yielding an equivalent reduction of measurement time, is the most promising among them. While the prospect to significantly reduce acquisition time by means of high multiband acceleration factors (M) appears tempting, signal quality parameters and the sensitivity to detect common RSNs with increasing M-factor have only been partially investigated up to now. In this study, we therefore acquired rs-fMRI data from 20 healthy volunteers to systematically investigate signal characteristics and sensitivity for brain network activity in datasets with increasing M-factor, M = 2 − 4. Combined with an inplane, sensitivity encoding (SENSE), acceleration factor, S = 2, we applied a maximal acceleration factor of 8 (S2×M4). Our results suggest that an M-factor of 2 (total acceleration of 4) only causes negligible SNR decrease but reveals common RSN with increased sensitivity and stability. Further M-factor increase produced random artifacts as revealed by signal quality measures that may affect interpretation of RSNs under common scanning conditions. Given appropriate hardware, a mb-EPI sequence with a total acceleration of 4 significantly reduces overall scanning time and clearly increases sensitivity to detect common RSNs. Together, our results suggest mb-EPI at moderate acceleration factors as a novel standard for fMRI that might increase our understanding of network dynamics in healthy and diseased brains.

## Introduction

Brain imaging with functional magnetic resonance imaging (fMRI) is currently the most widely applied technique to study the human brain on a systems level. Particularly, brain network imaging based on the coherence of ongoing blood oxygenation level dependent (BOLD) signal fluctuations, called resting-state fMRI (rs-fMRI), is a promising technique to illuminate system level alterations in neuropsychiatric disorders [1, 2]. Currently, echo planar imaging (EPI) [3] with its short acquisition times and heavy T2* weighting, i.e. high sensitivity towards the BOLD effect, represents the workhorse for almost all fMRI studies [4, 5]. Typical acquisition times for whole brain coverage amount to several seconds for a single imaging volume. Within the last few decades, partial Fourier [6] and parallel imaging [7–9] as well as sparse sampling techniques [10–12] achieved a considerable acceleration, allowing acquisition times of about two to three seconds for single volumes covering the whole brain with an isotropic spatial resolution of 3 mm. Even though these techniques are comparatively fast, we are still not able to fully examine all spatial and temporal aspects of large-scale brain networks. Further reduction in scan time by parallel imaging is hampered by the fact that higher acceleration factors increasingly shorten the EPI echo train, and thus also reduce the effective echo time. This is undesirable because an optimal BOLD contrast actually requires TE ≈ T2*. Taking into account the SNR decrease at longer echo times and the severe signal loss in areas with strong background susceptibility gradients as in temporal and orbitofrontal brain areas, echo times of 30 to 40 ms are most frequently used in fMRI applications at 3 T (11), even though T2* was found to be about 50 ms in wide areas of the human brain [13].

The recently introduced multiband (mb) or simultaneous multi-slice (sms) EPI technique [14] effectively shortens acquisition time without decreasing TE and, principally, without sacrificing SNR by simultaneous acquisition of multiple slices. Similar to parallel imaging, spatial encoding critically relies on the spatially varying sensitivities of RF receive coil arrays. The technique requires multiband RF pulses which achieve simultaneous excitation of multiple slices [15, 16], and reconstruction is most easily done by means of the SENSE algorithm [14, 17]. This method facilitates a considerable shortening of volume acquisition times because the number of simultaneously acquired slices, i.e. the multiband factor (M), directly translates into a reduced number of excitations and thus measurement time. Similar to parallel imaging the specified spatial resolution is preserved but there is no direct SNR penalty $∼1/\sqrt{R}$ due to a reduced number of acquisitions with acceleration factor R. However, depending on coil geometry, there may be a spatially dependent noise amplification with increasing M-factor which can be characterized by a geometry factor g [18], similar to parallel imaging.

Nunes et al. first demonstrated simultaneous multi-slice EPI in the human brain [19]. They found strong noise amplification because small distances between simultaneously excited slices cause an ill conditioned unaliasing problem. In order to mitigate this, they used the wideband technique [19, 20] where unipolar gradient blips in slice direction are used to introduce a shift between pixels in phase encoding (PE) direction. This assists the unfolding, but also causes an effective voxel tilt resulting in a significant blurring artifact. Setsompop et al. recently extended this technique introducing 'blipped-CAIPI' where alternating phase shifts similar to 'controlled aliasing in parallel imaging results in higher acceleration' (CAIPIRINHA) [21] are achieved by means of balanced gradient blips in slice direction [22]. Xu et al. systematically compared the noise enhancement and signal leakage between simultaneously excited slices in mb-EPI using slice acceleration factors up to 12 [23]. They concluded that acceleration factors up to eight can be used when blipped CAIPI is employed for aliasing control. In the mean time, simultaneous multi-slice EPI has also been combined with further acceleration techniques such as parallel imaging [24] and simultaneous echo refocusing (SIR) [25, 26].

Overall, mb-EPI sequences by now appear mature enough to be applied on a broader scale (see mb-EPI in human connectome project (HCP) [27]). A number of studies demonstrated an impressive potential enhancement of resting as well as task fMRI capabilities [24–26, 28–32]. However, only two studies exist which took a closer look on image quality by analyzing signal leakage at different M-factors [23] and performance of highly accelerated EPI combining mb and SIR in task fMRI [26]. The influence of increasing multiband factor at otherwise constant parameters was recently investigated by Xu et al. [23]. At least to our knowledge, no study has so far evaluated signal quality and performance of mb-EPI for rs-fMRI under realistic conditions using time efficient sampling at minimum TR and optimized flip angle. We therefore measured a group of healthy subjects considering the context of multichannel coils [33, 34], parallel imaging [9, 18, 35], simultaneous multislice acquisitions [23], as well as the presence of physiological noise [34, 36–39].

The aim of this study was, to closely evaluate the signal characteristics, i.e. SNR and g-factor related noise enhancement, as well as the resulting statistical measures of large-scale rs-fMRI networks in a cohort of 20 healthy volunteers using minimum TR and optimized flip angle. On the basis of our standard EPI protocol (M-factor, M = 1) with a 2-fold inplane SENSE acceleration (SENSE factor, S = 2), we established a multiband EPI sequence with up to 4-fold slice acceleration (M = 2, 3, 4), resulting in a maximum total acceleration of 8 (S2×M4). Each protocol covered the whole brain with an isotropic spatial resolution of 3 mm, and used the respective (almost) minimum repetition time and optimum flip angle to achieve most efficient sampling during the respective 7 min of acquisition time.

## Methods

### Subjects and Instrumentation

20 healthy subjects (11 female, age 24.6 ± 4.1 years) participated in this study and gave written consent after being informed about the objective and potential risks. The study was approved by the local ethics committee (Fakultät für Medizin, TU München, Munich, Germany) and was conducted according to the principles expressed in the Declaration of Helsinki. Experiments were performed on a Philips Ingenia 3 T scanner (Philips Healthcare, Best, The Netherlands) using the body coil for transmission and the standard 32 channel head coil for signal reception.

### Imaging Protocol

We used a mb-EPI sequence [14] with blipped-controlled aliasing [21, 22]. In order to compare the performance of mb-EPI acquisitions with different slice acceleration, rs-fMRI data were acquired for 7 min using four protocols with different M-factors and number of volumes (n), where the volume repetition times (TR) were near minimum and flip angles (α) were adjusted to yield maximum signal (Ernst angle). The subjects were instructed to lie as still as possible during scans, keep their eyes closed and think of nothing in particular. With respect to the protocols with different multiband factors, the scan order was permuted between subjects to avoid order effects on the results.

All investigated mb-EPI protocols used SENSE [9] with an acceleration factor 2 (S2) since this was shown to reduce susceptibility artifacts with minimal SNR degradation [35]. A matrix size of 64×64, a voxel size of 3×3×3 mm^3^ and 36 slices with 0.3 mm gap allowed whole brain coverage in the majority of subjects. The protocols with the different multiband factors are referred to as S2×M1, S2×M2, S2×M3 and S2×M4 in the following. We used M-factors of M = 1, 2, 3, 4, TR = 2000ms, 1000ms, 700ms, 520ms, α = 90°, 70°, 60°, 50°, resulting in a total of n = 210, 420, 610, 810 volumes. For these large multi-volume data sets with M ≥ 2, reconstruction took up to about 20 minutes depending on the data size. To avoid delays in the scan process, for M ≥ 2 raw data were saved on the scanner and the reconstruction was performed offline on a Windows PC using a dedicated reconstruction software based on the SENSE algorithm (developed with ReconFrame, GyroTools, Zürich, Switzerland). The reference experiment (S2×M1) was reconstructed at the scanner using standard software. Additionally, a T1w anatomical 3D-TFE data set with an isotropic spatial resolution of 0.7 mm and a FLAIR data set were acquired for each volunteer.

### Analysis of Image Quality and SNR

Data processing with regard to image quality and signal-to-noise ratio (SNR) used SPM8 (http://www.fil.ion.ucl.ac.uk/spm) and custom programs written in Matlab (MathWorks, Natick, Massachusetts, USA). Therefore, all data were converted into NIFTI format (.img/.hdr). For all mb-EPI time series from each subject, motion correction using the SPM8 realignment procedure with standard parameters and quadratic detrending with a custom procedure were performed.

Anatomical T1w TFE data were segmented into gray and white matter in native space and coregistered to the mean EPI volume generated during realignment for each subject. GM and WM masks were generated from these segments using a threshold of 0.75 for the probability maps generated by SPM8.

The temporal SNR (SNR(t)) [35] was calculated voxelwise for all four multiband EPI time series of each subject by calculating the quotient of the mean 〈*S*(*t*)〉 and the standard deviation of the signal *σ*(*S*(*t*)) across the time course according to

$$SNR(t)=\frac{\langle S(t)\rangle }{\sigma (S(t)}\;\text{where}\;\sigma (S(t))=\sqrt{\frac{1}{n-1}\sum_{i=1}^{n}(S_{i}-{\langle S(t)\rangle }^{2}}$$ (1)

SNR(t) was calculated for the full (SNR_full_(t)) (n = number of volumes) as well as for truncated time series of 210 volumes (SNR_210_(t)) which were also used for analysis with regard to resting state networks.

The gold standard to assess structured, spatially varying noise due to residual aliasing in parallel imaging consists in the calculation of maps of the geometry factor g according to [18]

$$g=\frac{SNR_{full}}{SNR_{acc}\;\sqrt{R}}$$ (2)

where SNR_full_ and SNR_acc_ denote the (temporal) SNRs of the fully sampled and of the accelerated images, respectively, and R is the acceleration factor. Simultaneous excitation and acquisition of multiple slices as performed in mb-EPI does not per se introduce an SNR penalty which is related to the achieved acceleration. Nevertheless, structured noise due to residual aliasing has been described as a kind of signal leakage characterized by a leakage factor L [23]. Since in this work we used simultaneous multiband excitation in combination with parallel imaging, and also reduced TR and flip angle to achieve most efficient sampling for resting fMRI experiments, we calculated apparent g-factor maps according to

$$g_{app}=\frac{SNR_{full,M=1}}{SNR_{acc,M>1}}$$ (3)

in order to assess the increase of structured noise with increasing multiband factor.

### Statistical Analysis of Resting Network

The statistical analysis was performed once on the full datasets of M-factors M1 to M4 (*full analysis*), resulting from time-series of equal acquisition duration (420 sec) but with increasing number of volumes (M1: 210 volumes to M4: 810 volumes). In order to also assess the reliability of statistical results in the case of equal numbers of volumes (i.e. decreasing acquisition time with increasing M-factor), the statistical analysis was also carried out on each M-factor’s subsets of the first 210 volumes (*truncated analysis*).

FMRI data processing was performed using FEAT (FMRI Expert Analysis Tool) Version 6.00, as part of FSL (FMRIB's Software Library, www.fmrib.ox.ac.uk/fsl). Spatial pre-processing comprised motion correction using MCFLIRT (motion correction FLIRT (FMRIB’s linear image registration tool)) [40], non-brain removal using BET (brain extraction tool) [41], spatial smoothing using a Gaussian kernel of FWHM (full width at half maximum) 6.0mm, grand-mean intensity normalization of the entire 4D dataset by a single multiplicative factor and highpass temporal filtering (Gaussian-weighted least-squares straight line fitting with σ = 62.5sec). Registration to the standard MNI 152 brain (spatial resolution 2 mm) was carried out using FLIRT [40, 42].

We performed rs-fMRI analysis separately for both approaches (*full* and *truncated* data sets) using Probabilistic Independent Component Analysis (pICA) [43] as implemented in MELODIC (Multivariate Exploratory Linear Decomposition into Independent Components) Version 3.14, as part of FSL (FMRIB's Software Library, www.fmrib.ox.ac.uk/fsl). The preprocessed datasets of all subjects (n = 20) and multiband factors (M1 to M4) were temporally concatenated, whitened and projected into a 70 dimensional subspace using Principal Component Analysis. The whitened observations were decomposed into sets of vectors describing signal variations across the temporal domain, i.e. time courses, the session/subject domain and across the spatial domain (brain maps) by optimizing for non-Gaussian spatial source distributions using a fixed-point iteration technique [44]. Estimated component maps were divided by the standard deviation of the residual noise and thresholded by fitting a mixture model to the histogram of intensity values [43].

The resulting set of spatial maps from this group-average analysis was used to generate subject-specific versions of the spatial maps, and associated time series, using dual regression [45, 46]. First, for each subject, the group-average set of spatial maps was regressed (as spatial regressor in a multiple regression) into the single subject's 4D space-time dataset. This resulted in a set of subject-specific time series, one per group-level spatial map. Next, those time series were regressed as temporal regressors, again in a multiple regression, into the same 4D dataset, resulting in a set of subject-specific spatial maps, one per group-level spatial map. Functionally relevant components were selected by visual inspection referring to an established baseline set of resting state networks [47]. We then tested for differences in spatial extent (factor: number of voxels) and stability of networks (factor: peak z-score) across M-factors (factor: M) in a repeated measures ANOVA-model and post-hoc paired-t-tests using FSL's randomized permutation-testing tool using 5000 permutations (p<0.001). Tests were separately performed on the full and truncated dataset.

## Results

### Image Quality and SNR

**Fig 1:**
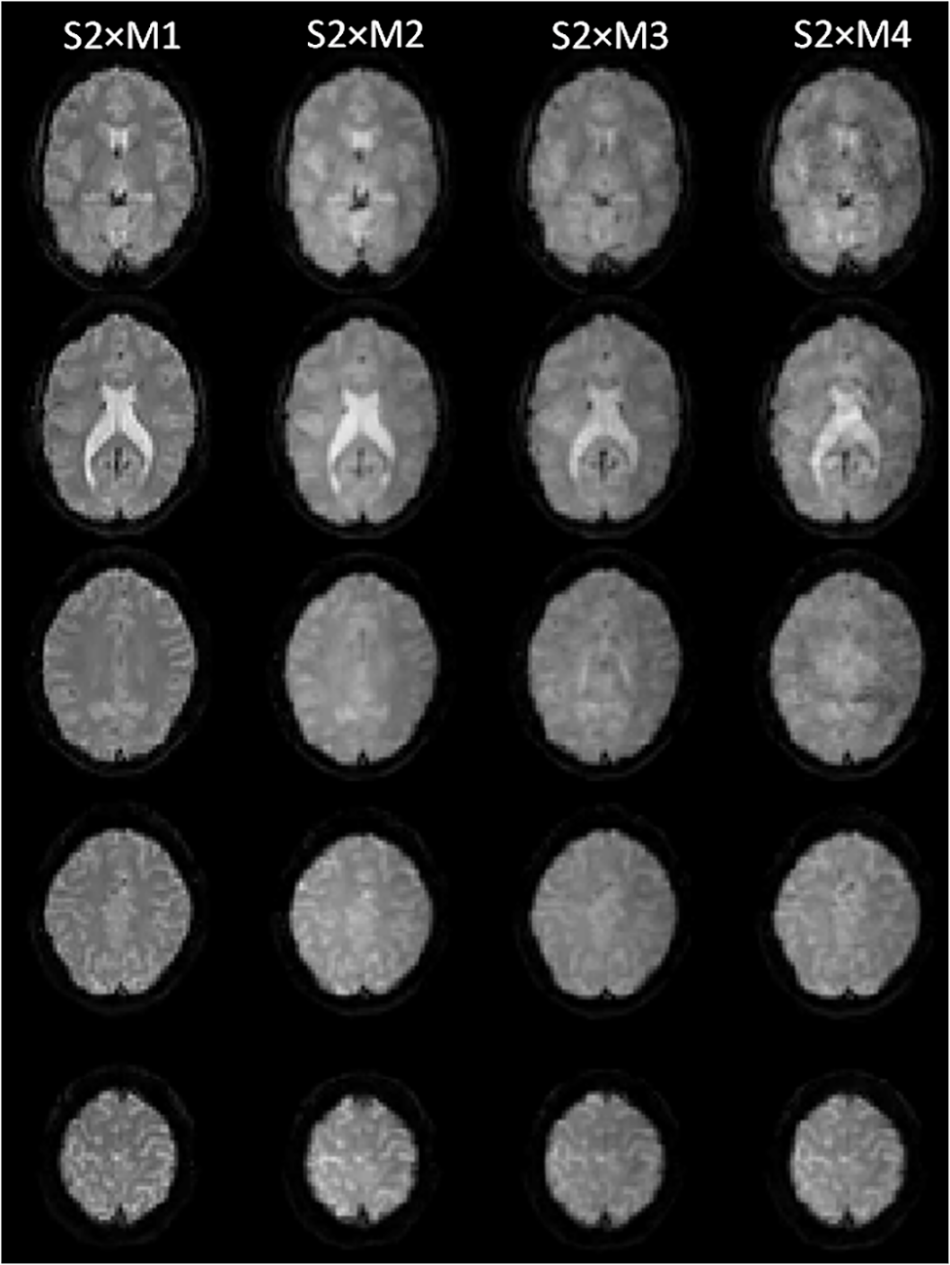
Selected slices of unprocessed EPI data sets for different M-factors from one subject.

**Fig 2:**
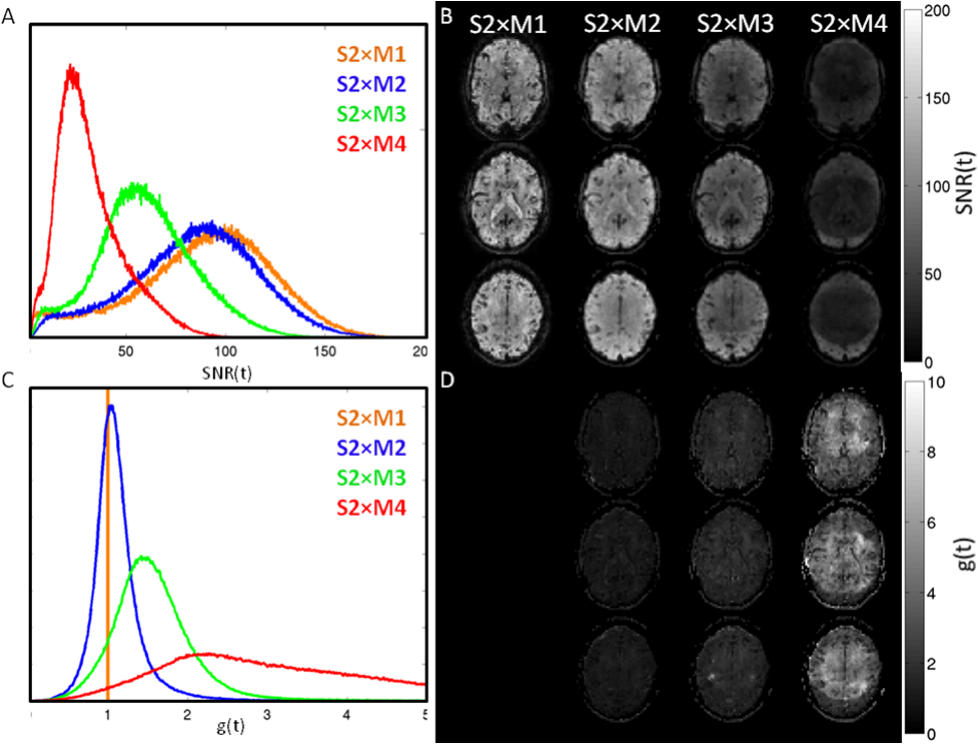
Histograms of temporal SNR(t) (A) and apparent g-factor g_app_(t) (C) as well as selected slices of temporal SNR(t) (B) and apparent g-factor maps (D) for different M-factors of one subject. The histograms were obtained by averaging the respective whole brain histograms of all 20 subjects for different M-factors.

Fig 1 shows selected slices from a single volume of one volunteer for all M-factors. On a first glance, the images appear quite similar. However, a closer look reveals that the images with increasing M-factor look somewhat degraded. A quantitative evaluation is presented in Fig 2 where histograms of temporal SNR (Fig 2A) and apparent g-factor (Fig 2C) are shown together with the respective maps (Fig 2B and 2D) of the same slices as in Fig 1. The histograms in Fig 2 represent whole brain data averaged across all 20 subjects. Fig 2A clearly illustrates that the temporal SNR of experiment S2×M2 is almost equal to the data from the reference experiment S2×M1, and that also the corresponding apparent g-factor (Fig 2C) is almost symmetrically distributed around 1. This impression is visually reflected by the similar image quality of SNR(t) for the standard S2×M1 and the S2×M2 experiment in the first and second column of Fig 2B as well as the corresponding g_app_-map in the second column of Fig 2D. With increasing M-factor (S2×M3 and S2×M4), a drop in temporal SNR (Fig 2A and 2B) as well as an increase in g_app_ (Fig 2C and 2D) occurs. In Fig 2D (3rd column, bottom row), one can also recognize focal spots of noise enhancement which occurred randomly across subjects and affected between four and ten slices at S2×M3. Strong, weak or no focal noise enhancement was seen in eleven, six and three subjects, respectively. A severe drop of temporal SNR (Fig 2A and 2B) and increase of g_app_(t) (Fig 2C and 2D) appears in experiment S2×M4. This is due to a massive increase of structured noise especially in the center of the brain which is obvious in temporal SNR (Fig 2B, 4th column) as well as g_app_(t) (Fig 2D, 4th column) maps.

Table 1 summarizes the subject averages of temporal SNR for *truncated* and *full* data as well as the apparent g-factors which were derived from SNR_full_(t). SNR values are tabulated for GM and WM separately, which demonstrates that the temporal SNR is generally higher in WM than in GM except for experiment S2×M4. SNR_full_(t) and SNR_210_(t) both show a similar decrease for higher multiband factors. For S2×M2, SNR_full_(t) and SNR_210_(t) amount to 93% and 94% of the reference experiment S2×M1 which decreases to 67% and 68% for S2×M3 and goes down to 36% for S2×M4.

**Table 1:** Subject averages (mean ± stddev) of temporal SNR and apparent g-factor (g_app_) for different multiband factors as determined in GM and WM VOIs.

	S2×M1		S2×M2		S2×M3		S2×M4	
	GM	WM	GM	WM	GM	WM	GM	WM
**SNR**_**full**_ **(t)**	83.2±8.5	98.8±9.2	78.1±9.2	90.2±9.2	58.6±7.0	60.2±7.1	33.4±3.8	28.7±3.6
**SNR**_**210**_ **(t)**	83.2±8.5	98.8±9.2	79.1±8.5	90.9±8.9	59.8±6.3	61.1±6.9	34.2±3.7	29.1±3.6
**g**_**app**_	1.00	1.00	1.08±0.09	1.12±0.06	1.46±0.20	1.73±0.20	2.74±0.32	3.97±0.32

All measures were derived from realigned and detrended time series data. Temporal SNR(t) (see Eq (1)) was calculated from the full time series (SNR_full_; T_acq_ = 7 min) as well as from truncated time courses of 210 volumes (SNR_210_). The apparent g-factor g_app_ (see Eq (3)) was determined from the full time series. The mean VOI size (number of voxels, mean ± stddev) in GM and WM was 20346±1513 and 14156±1329, respectively.

### Statistical Analysis of Resting Networks

**Fig 3:**
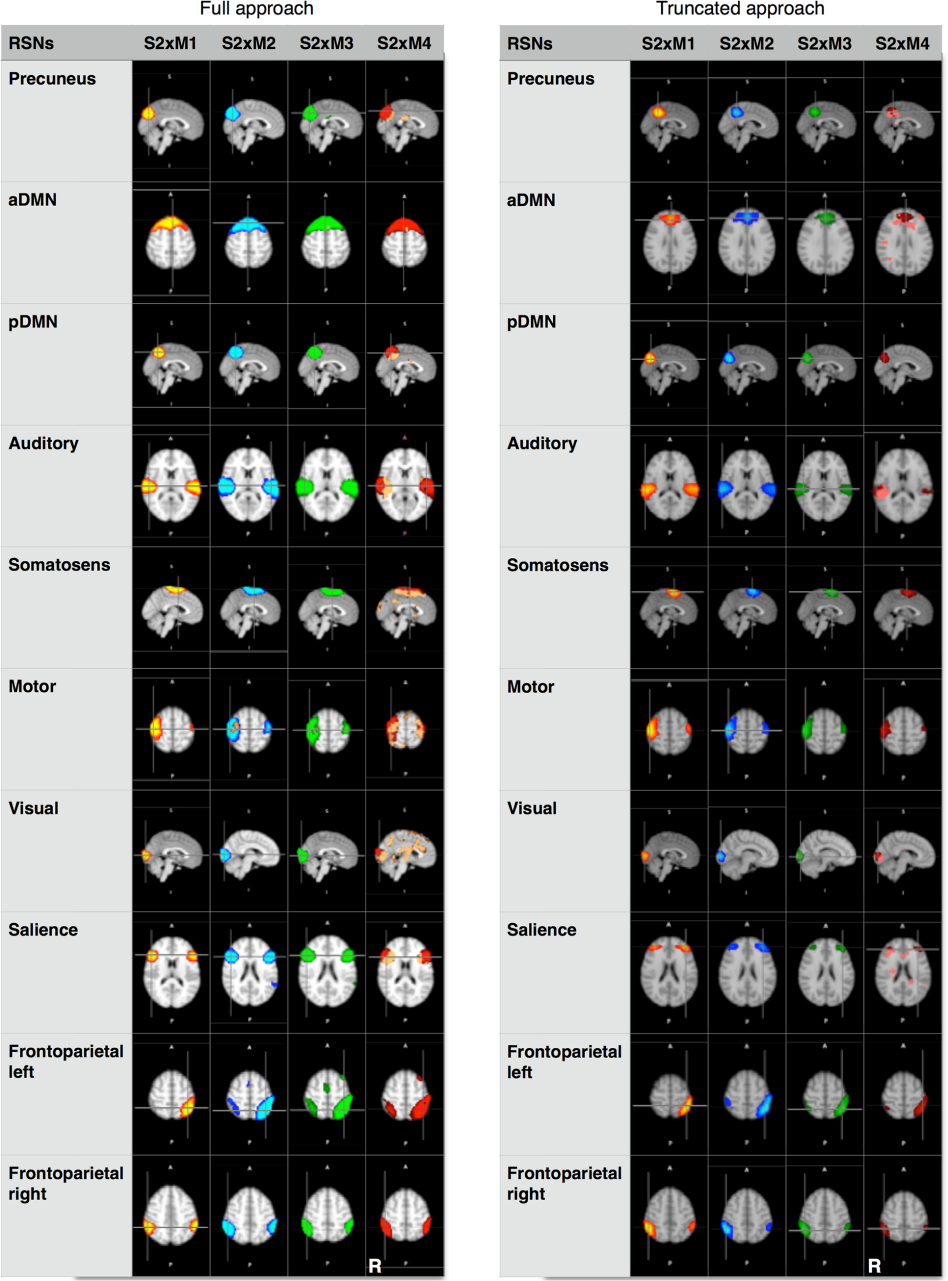
Spatial representation of the ten representative resting state networks. Networks were obtained from full (left panel) and truncated (right panel) data sets for different M-factors (S2×M1: yellow, S2×M2: blue, S2×M3: green; S2×M4: red). Please note the strong variability of networks at M4 indicated by voxel-wise differences at p<0.05 corrected for TFCE (orange voxels in red column).

In addition to qualitatively and quantitatively evaluating the temporal and spatial signal behavior of the multiband EPI time series, we here explored the sensitivity of the four mb-EPI protocols to detect functional brain networks on the basis of coherent BOLD-signal fluctuations. For each approach separately, i.e. the *full* and *truncated* dataset, we first identified the 10 most relevant resting state networks (RSN) using pICA and subjected the 4 M-factor maps of each network to a voxel-wise ANOVA to test for differences in spatial extent. Fig 3 presents the group average maps (1-sample t-test) of the 10 RSNs for each M-factor of the *full* (left column) and *truncated* analysis (right column). Additionally, voxel-wise differences in the stability of the RSNs compared to M1 are plotted in orange for each network (p<0.05, corrected for threshold-free cluster enhancement (TFCE)). Up to M3, the networks are remarkably stable across M-factors for the *full* as well as the *truncated* data sets. Only for M4, the ANOVA illustrates increased variability in almost all networks (orange voxels in green networks of M4), even in the *full* approach with 4 times the amount of data points. It is important to note that the truncated S2×M3 mb-EPI time series is sensitive enough to detect all relevant RSNs in only about one third of the total acquisition time (acquisition time for 210 volumes S2×M3: 147 sec vs. S2×M1: 420 sec).

**Fig 4:**
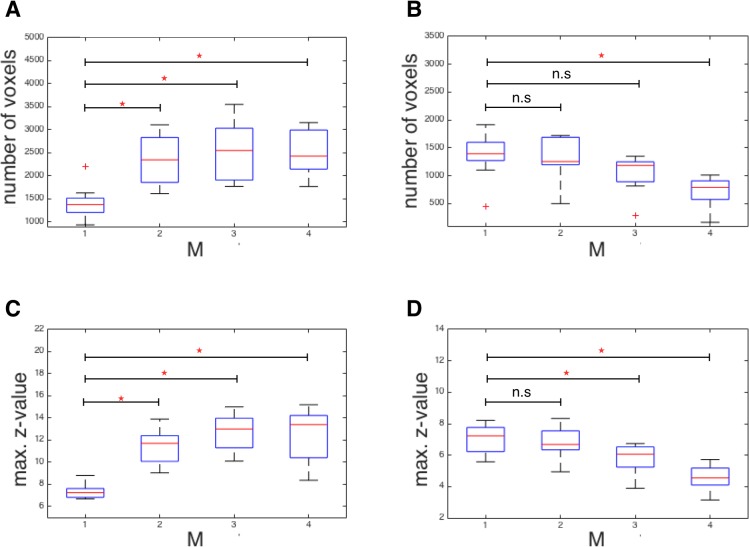
Box plots of the total number of voxels (A, B) and maximum z values (C, D) of full (A, C) and truncated (B, D) data sets averaged across all subjects (n = 20) and ten investigated RSNs. Significant differences are marked by an asterisk (p < 0.001, repeated-measures ANOVA).

Finally, we aimed at quantifying the differences in network stability in terms of spatial extent (number of voxels in a RSN with z > 3) and signal amplitude (peak z-score in a RSN). The box plots in Fig 4 show for each M-factor (i) the total number of voxels across all networks (Fig 4A/4B: full/truncated dataset) and (ii) the peak z-score across all networks (Fig 4C/4D: full/truncated). We separately subjected these two parameters to a repeated-measures ANOVA testing for significance at p<0.001. In the *full* datasets we found a significantly improved network sensitivity both with respect to spatial extent and maximum z-score for all M-factors in comparison to M1 (Fig 4A/4C). However, in M4, the peak z-score has a wide error margin reaching partly down to the range of M1. Fig 4B/4D shows the results for the *truncated* datasets with an identical number of 210 volumes acquired in a decreasing amount of time. These plots indicate that in contrast to the increased stability with higher M-factors as identified in the full dataset, the number of voxels and maximum z-values are rather decreased for the truncated data due to shorter acquisition time. Particularly, M4 shows a significantly decreased sensitivity for detecting RSNs in terms of spatial extent (Fig 4B) and maximum z-values (Fig 4D). However, as the results of M2 indicate, it seems possible to detect all relevant RSN with almost equal sensitivity in only half the acquisition time. Even with M3, we detected all relevant RSNs (see Fig 4, right column) in full spatial extent, however with significantly decreased peak z-value.

## Discussion

In this study, we investigated four different mb-EPI protocols with regard to the image quality, temporal SNR, structured noise due to residual unfolding artifacts and, finally, their sensitivity to detect common RSN from rs-fMRI. Taken together, a moderate slice acceleration factor M2 with an appropriately reduced TR and flip angle yielded a data quality essentially equal to the reference experiment with 2-fold in plane acceleration (SENSE), and thus clearly improved statistical results of functional network analyses or alternatively facilitated bisection of acquisition time. Higher slice acceleration, i.e. M-factors, in combination with 2-fold in plane acceleration increasingly reduced data quality with significant structured noise enhancement at M4.

### Image Quality and SNR

Meaningful SNR comparisons of imaging protocols are difficult in the context of multichannel coils [33, 34], parallel imaging [9, 18, 35], simultaneous multislice acquisitions [23], and especially in the presence of physiological noise in fMRI time series [34, 36–39]. In this work, we evaluated the temporal SNR and apparent g-factor maps in order to characterize data quality with regard to the intended functional imaging application. Comparing a standard EPI sequence with a SENSE acceleration factor of 2 (reference protocol S2×M1 with M-factor M = 1) to the S2×M2 acquisition (M = 2, total acceleration factor 4), the temporal SNR only showed a negligible decrease, and there was also no structured noise enhancement visible in the apparent g-factor map (see Fig 2). With S2×M3 (M = 3, total acceleration factor 6), there was a significant loss in temporal SNR of about 33% and in several slices of some subjects there were focal spots of noise enhancement visible in the spatial maps of temporal SNR (see Fig 2). At S2×M4 (M = 4, total acceleration factor 8), finally, the SNR loss of about 64% was dramatic and the apparent g-factor maps also showed strong noise enhancement in the center of the brain. This fits with results of Xu et al. [23] who found increasing g-factors with increasing mb-factors. Especially, their g-factor histograms (Fig 2 in ref.[23]) nicely resemble ours (see Fig 2C) when taking into account the total acceleration which is 2 times M in our case, since we additionally used a SENSE in plane acceleration factor of 2.

Interestingly, the temporal SNR is very similar irrespective of whether it is determined from the full (SNR_full_(t)) or from the truncated (SNR_210_(t)) data sets (see Table 1, Fig 3). This behavior can be explained by the dominance of physiological noise [37] which also fits with the finding that SNR in GM is lower than in WM except for the S2×M4 experiment where structured noise due to residual unfolding artifacts dominates especially in the center of the brain (see Table 1, Fig 2).

### Implications for Resting State fMRI

Overall, our analyses revealed a clearly increased sensitivity to detect RSNs with increasing M-factor up to M3 that finally decreased again with M4. Group analysis of the full mb-EPI times series showed an increased extent and maximum z-value (see Fig 4) for ten representative RSNs which agrees with results of Feinberg et al. [25] and Chen et al. [26] who demonstrated increased functional sensitivity at higher sampling rates. Upon visual inspection, all 10 RSNs can be detected in the full as well as in the truncated datasets, yet the ANOVA reveals spurious signals in almost all networks for M4. Quantitative analysis supports this observation, revealing significantly increased sensitivity for spatial extent and maximum z-values for M2 and M3 but not for M4 in the full dataset. This reflects the fact that structured noise due to residual unaliasing artifacts is critically enhanced which is prohibitive for functional imaging applications. Statistical analysis of the truncated data sets (n = 210 volumes) indicates that sensitivity for the 10 RSNs is almost fully preserved for the moderately accelerated S2×M2 experiment whereas there is a clear trend towards reduced sensitivity for S2×M3. At S2×M4, finally, the extent and maximum z-values are significantly reduced und the variability of spatial maps across subjects is likewise significant (see Fig 4). Interestingly, the analysis of the truncated datasets indicates that mb-EPI can detect most common RSNs of slow frequency fluctuating BOLD-signals in short acquisition times down to 2.45 minutes (M3). Since Feinberg et al. [25] and Chen et al. [26] demonstrated increased sensitivity to functional activation at higher sampling rates we assume that these results are equally applicable to task fMRI.

## Conclusion

Our results suggest that the multiband technique, allowing simultaneous acquisition of multiple slices, suffers from a minimal or tolerable SNR penalty for an M-factor of 2 when it is combined with a SENSE factor of 2 (total acceleration of 4) as in the current study. The analysis of subsets of only 210 volumes suggests that the use of the mb-EPI technique may even permit to bisect fMRI acquisition times e.g. in clinical studies. Data acquired with an M-factor of 4 (total acceleration of 8) on the other hand are significantly impaired by structured noise enhancement which in our opinion is prohibitive for fMRI applications. However, no general advice can be given with respect to an M-factor of 3 (total acceleration of 6). In this case, the statistical yield compared to M = 2 is minimal at best but there is some focal noise enhancement at least in some subjects which might compromise fMRI results. However, when a higher temporal resolution is urgently needed, it might be a viable option. In any case, future developments in coil design and reconstruction techniques might soon be able to shift the border with respect to usable acceleration factors.
